# Synthesis and Antiradical/Antioxidant Activities of Caffeic Acid Phenethyl Ester and Its Related Propionic, Acetic, and Benzoic Acid Analogues

**DOI:** 10.3390/molecules171214637

**Published:** 2012-12-10

**Authors:** Luc M. LeBlanc, Aurélie F. Paré, Jacques Jean-François, Martin J. G. Hébert, Marc E. Surette, Mohamed Touaibia

**Affiliations:** Department of Chemistry and Biochemistry, Université de Moncton, Moncton, NB E1A 3E9, Canada; E-Mails: LC280974@dal.ca (L.M.L.); aurelie.pare@usherbrooke.ca (A.F.P.); jacques.jean-francois@umoncton.ca (J.J.-F.); emh3363@umoncton.ca (M.J.G.H.); marc.surette@umoncton.ca (M.E.S.)

**Keywords:** CAPE, caffeic acid, 3-(3,4-dihydroxyphenyl) propanoic acid esters, 2-(3,4-dihydroxyphenyl) acetic acid esters, 3,4-dihydroxybenzoic acid esters, antioxidant

## Abstract

Caffeic acid phenethyl ester (CAPE) is a bioactive component isolated from propolis. A series of CAPE analogues was synthesized and their antiradical/antioxidant effects analyzed. The effect of the presence of the double bond and of the conjugated system on the antioxidant effect is evaluated with the analogues obtained from 3-(3,4-dihydroxyphenyl) propanoic acid. Those obtained from 2-(3,4-dihydroxyphenyl) acetic acid and 3,4-dihydroxybenzoic acid allow the evaluation of the effect of the presence of two carbons between the carbonyl and aromatic system.

## 1. Introduction

From ancient times to nowadays, whether on an empirical or rational basis, natural-based molecules have long been used as drugs or drug leads. Several small molecules available worldwide on the drug market can be traced back to or were inspired by natural products [1]. In the ongoing search for new therapeutic compounds, phenolic acids, which are widely distributed in plants [2], are very important because of their interactions with several biological targets. Many phenolic acids are linked to cell wall components such as arabinoxylans and proteins [3,4] and are used as antioxidants in various food ingredients, such as fatty acids and oils [5,6]. Other uses of phenolic acids include the fortification of diets in order to provide antimutagenic, antiglycemic, and antioxidative benefits. Such general characteristics of phenolic acids can be exploited to develop health foods [7]. 

Caffeic acid, the main representative of the hydroxycinnamic and phenolic acids, is found in many plants as simple derivatives such as glycosides, amides, esters and sugar esters. Caffeic acid phenylethyl ester (CAPE), one of the most active compounds in propolis, is the perfect illustration of a natural compound exhibiting diverse biological activities. Numerous studies have shown that CAPE has antioxidant, anti-inflammatory, antitumoral, and antifungal activities [8,9,10]. CAPE’s strong antioxidant effect can result from transcriptional inhibition of NF-κB and a diminished expression of pro-inflammatory genes [9,11]. Free radical scavenging, metal ion chelation and inhibition of specific enzymes that induce free radical or lipid peroxidation are acknowledged as the diverse mechanisms used by CAPE in its antioxidant activity [12,13]. Based on these promising results, several groups have started to explore strategies for the synthesis of caffeic acid or CAPE analogues with improved biological activities.

The antioxidant activities of caffeic acid and CAPE analogues are based on structural factors as well as the medium used to assay these activities. Son and Lewis, using an aqueous dispersion of linoleic acid (lipid peroxidation assay), showed that free radical scavenging activity was dependent on the presence and the number of catechol and hydroxyl groups present, and also on the number of H donating groups. In the emulsion medium used for the assay, the hydrophobicity of the analogues was also an important factor in determining their activity [14]. In addition to these known structural features, it was further demonstrated that phenolic acids bearing a carbonyl group separated from the aromatic ring were more active (cinnamic acid, caffeic acid) than their counterparts where the carbonyl is directly linked to the aromatic ring (benzoic acid) [15]. 

Jayaprakasam and co-workers synthesized a series of caffeic acid analogues with variable alkyl chain lengths. The antioxidant activity of these compounds was measured by the lipid peroxidation assay. In the lipophilic medium used for this test, the more lipophilic, long-chain alkyl esters (C_16_–C_22_) were the most potent antioxidants. Compounds with medium alkyl chain length (C_4_–C_8_) were less active (<20% inhibition) compared to caffeic acid (>80% inhibition) [16]. To further assess the structural parameters related to antioxidant activity, Silva and co-workers synthesized three alkyl (methyl, ethyl, and propyl) derivatives of caffeic and dihydrocaffeic acids. The radical scavenging activity of both classes of molecules was evaluated with the 2,2-diphenyl-1-picrylhydrazyl (DPPH) assay. With the exception of caffeic acid, most of the tested compounds, including dihydroxycaffeic acid, showed better radical scavenging than α-tocopherol, a reference compound. The ethylene moiety didn’t influence radical scavenging activity since dihydrocaffeic acid was more potent than caffeic acid. Increasing the alkyl chain length in a modest fashion, from methyl to propyl, has no significant effect on radical scavenging activity [17]. 

Using a Knoevenagel condensation, Zhang and co-workers synthesized a caffeic acid derivative, namely caffeic acid 3,4-dihydroxyphenethy ester, in high yield. Radical scavenging activity was evaluated with the DPPH assay and it appears that the 3,4-dihydroxyphenethyl ester analogue shows strong radical scavenging activity [18]. Caffeic acid is prone to oxidative dimerization [19]. In natural compounds such as caffeoylquininic acid, where two or three caffeoyl moieties might be present, such dimerization could influence the antioxidant activity of the molecules. To address this issue, Saito and co-workers synthesized 6 regio- and stereoisomers of dicaffeoyloxycyclohexanes and 2,4-di-*O*-caffeoyl-1, 6-anhydro-β-D-glucose as model compounds to evaluate the effect of intramolecular coupling between two adjacent caffeoyl residues. The radical scavenging activity of these compounds was evaluated with the DPPH and ABTS (2,2'-azino-bis(3-ethylbenzothiazoline-6-sulphonic acid) assays and compared against cyclohexyl caffeate. The results underlined the importance of the orientation or the distance between two adjacent caffeoyl moieties on antioxidant activity. Thus, the radical scavenging activity was at its lowest with compounds where the two caffeoyl residues were too far apart to interact. The highest activity was shown by the compound where the diaxial conformations of the two caffeoyl residues maximize their interaction [20].

Owing to the biological significance of caffeic acid analogues, we report herein the synthesis of phenethyl and phenpropyl esters of caffeic acid, 3-(3,4-dihydroxyphenyl) propanoic acid 2-(3,4-dihydroxyphenyl) acetic acid, and 3,4-dihydroxybenzoic acid. The antioxidant activity of the synthesized derivatives was investigated using two tests. Antioxidants employ two major strategies to deactivate radicals: hydrogen atom transfer (HAT) and electron transfer (ET) [21]. To evaluate which mechanism was favored by our test compounds we used two different tests. The DPPH assay was used to evaluate the free radical scavenging activity of the test molecules by an ET reaction. The APPH (2,2'-azobis(2-amidinopropane) dihydrochloride) assay was used to determine the antioxidant activity via an HAT mechanism mainly by assessing the prevention of lipid peroxidation in an emulsion system [22]. The present structure-activity relationship (SAR) study examines the effect of hydrogenation and the shrinking of the linker between the catechol moiety and the carbonyl on antioxidant activity. The lengthening of the linker from phenethyl to phenpropyl is also investigated.

## 2. Results and Discussion

### 2.1. Synthesis

**Scheme 1:**
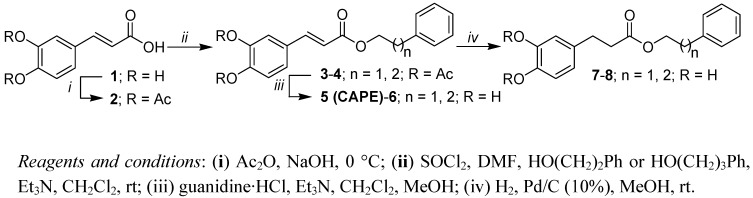
Synthesis of caffeic acid and 3-(3,4-dihydroxyphenyl) propanoic acid esters.

The syntheses of the phenethyl and phenpropyl esters of caffeic acid as well as the corresponding 3-(3,4-dihydroxyphenyl) propanoic esters are depicted in Scheme 1. Commercially available caffeic acid (**1**) is treated with sodium hydroxide and acetic anhydride at 0 °C. Recrystallization of the crude product from ethanol provides pure diacetylcaffeic acid (**2**) [23] in good yield. Esters **3** [24] and **4** were synthesized from 2-phenylethanol or 3-phenylpropanol and acetylated caffeic acid **2**. The conversion of **2** into the corresponding carboxylic chloride was achieved by the Vilsmeier**-**Haack adduct [25] derived from thionyl chloride with *N,N*-dimethylformamide (DMF) as catalyst. As shown in Scheme 1, phenethyl (CAPE) and phenpropyl esters of caffeic acid **5** [24] and **6**, were efficiently obtained by base-induced de-*O*-acetylation. Subsequently, esters **5** and **6** were converted into corresponding 3-(3,4-dihydroxyphenyl) propanoic esters **7** and **8** by hydrogenation over a palladium catalyst. 3-(3,4-Dihydroxyphenyl) propanoic acid (dihydrocaffeic acid, **9)**, was also obtained by hydrogenation of caffeic acid under the same conditions.

**Scheme 2:**
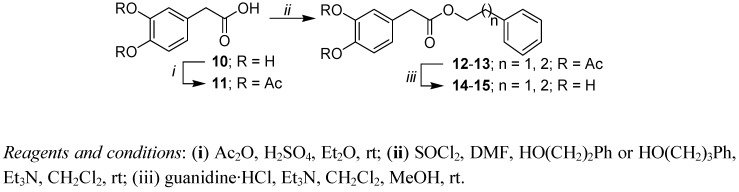
Synthesis of 2-(3,4-dihydroxyphenyl) acetic acid esters.

As shown in Scheme 2, 2-(3,4-dihydroxyphenyl) acetic phenethyl and phenpropyl esters **14**, **15** in which one carbon separates the carbonyl group from the catechol moiety were also synthesized. Synthesis began with the protection of the two hydroxyl groups of the commercially available 2-(3,4-dihydroxyphenyl) acetic acid (3,4-DOPAC, **10**) by acetate groups in acetic anhydride in the presence of sulfuric acid. As with **3** and **4**, 2-(3,4-dihydroxyphenyl) acetic esters **14** and **15** were obtained after the conversion of **11** into the corresponding carboxylic chloride, reaction with 2-phenylethanol or 3-phenylpropanol in presence of triethyl amine, and finally by a base-induced de-*O*-acetylation. 

**Scheme 3:**
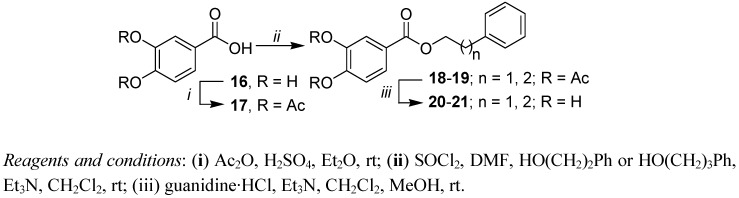
Synthesis of 3,4-dihydroxybenzoic acid esters.

Commercially available 3,4-dihydroxybenzoic acid (3,4-DHB, **16**) was used for the synthesis of caffeic acid ester analogs **20**, **21** in which no carbon separates the carbonyl group from the catechol moiety. The same strategy for the synthesis of **14** and **15** is used for the preparation of these esters (Scheme 3).

### 2.2. Free Radical Scavenging Activity

The DPPH assay was used to assess the free radical scavenging activity of our synthesized compounds. Namely we investigated their capacity to block the formation of the purple DPPH radical (DPPH^.^) by reducing these radicals to the corresponding yellow hydrazine. Special attention was required for the preparation of the control (DPPH reagent + ethanol without test compounds). The antiradical activities of all compounds are shown in Table 1. All of the IC_50_ are calculated relative to the control. Magalhaes *et al*. have shown that in this case the percentage of radical scavenged is dependent on the initial concentration of the radical DPPH [26]. All of the tested compounds incorporated basic structural features favoring antioxidant activity: catechol groups/hydroxyl groups and the presence of carbonyl groups. In this study we tried to evaluate the effect of other structural parameters present in caffeic acid/CAPE: the ethylene moiety, the length of the linker attaching the carbonyl to the catechol moiety and the length of the linker between the phenethyl group and the carbonyl. It appears that for a certain length of the linker between the catechol group and the carbonyl (2C with a double bond, an ethylene moiety) the free radical scavenging activity is better. Caffeic acid(**1**), CAPE (**5**) and **6** all bear an ethylene moiety and are more active (IC_50_ = 10–16 µM) at scavenging radicals than their counterparts **7** and **8** (IC_50_ = 23–25 µM) lacking the double bond but still retaining the 2C between the carbonyl and the catechol ring, so the ethylene moiety is important, but not a determining feature dictating radical scavenging activity, as illustrated by 3,4-DOPAC **10** and dihydrocaffeic acid (**9**) which, despite lacking the ethylene moiety, are the most active of the tested compounds (IC_50_ = 7–8 µM). While assessing the contribution of the ethylene link in antioxidant activity, Jayaprakasam and co-workers obtained similar results by showing that dihydrocaffeic acid which lacks the ethylene link was more active than caffeic acid itself [16]. 

**Table 1:** Free radical scavenging activity of test compounds.

Compound	IC_50_ (μM)	Compound	IC_50_ (μM)
1	15.3	10	7.1
5	16.5	14	18.1
6	11.9	15	20.3
9	7.8	16	43.7
7	24.6	20	20.4
8	23	21	9.6

Direct attachment of the carbonyl group on the catechol ring negates or decreases radical scavenging activities as reported earlier by other groups [27]. DHB **16**, whose carbonyl group is directly linked to the aromatic ring, has the lowest free radical scavenging activity (IC_50_ = 44 µM) of all the tested compounds. By comparison 3,4-DOPAC **10**, whose carbonyl has been moved one C away from the aromatic ring, is much more potent (IC_50_ = 7 µM). A further increase to 2 C atoms away from the aromatic ring doesn’t bring about any significant change (hyCaf Ac (**9**): IC_50_ = 7.8 µM).

As to the influence of the link between the carbonyl and the phenethyl, the patterns are less obvious. The analogues DHB (compound **16**), **20** and **21** all lack the ethylene moiety and their carbonyl is directly linked to the catechol ring. In this particular case DHB **16** still showed poor antiradical activity. Compounds **20** and **21** showcasing a phenyl group separated from the carbonyl by a linker of 2 and 3 C atoms, respectively, were much more potent. Lengthening the linker between phenethyl and carbonyl from 2 C (compound **20**) to 3 C atoms (compound **21**) increased the activity by almost two-fold. 

It appears that the ethylene group gains significance when there are 1 to 2 C atoms between the carbonyl and the catechol. Thus compounds **7**, **8**, **14** and **15** lacking the ethylene are less active at scavenging radicals than CAPE (**5**) and compound **6** which contain the ethylene moiety. In such a case the presence of the phenethyl group seems to be less relevant as illustrated by the similar IC_50_ of CAPE(**5**) and caffeic acid (**1**). They both have the ethylene moiety but CAPE (**5**) also has a phenethyl group. The influence of the phenethyl group is more important when the carbonyl is directly linked to the catechol ring. Based on these results it seems that the most potent radical scavengers in this series of CAPE (**5**) analogues are molecules bearing catechol rings and a carbonyl group separated from the aromatic ring by 1–2 C atoms.

### 2.3. Antioxidant Activity

Antioxidant activity mainly involves two major mechanisms: electron transfer (ET) and hydrogen atom transfer (HAT). Thus it is of interest to evaluate which one of these mechanisms is favored by our test compounds. Carotenoids are relatively poor quenchers of peroxyl radicals and are exceptionally active at quenching singlet oxygen, whereas phenolic compounds are active against peroxyl radicals and ineffective against singlet oxygen [21]. Among the effects produced *in vivo* by radicals, lipid peroxidation is a serious one. Lipid peroxides resulting from radical action can alter the membrane fluidity and modify the function of membrane proteins. In turn these lipid peroxides can generate peroxyl radicals leading to toxic end-products such as malondialdehyde [22].

Peroxyl (ROO•) scavenging activity assesses the capacity of an antioxidant to quench these radicals by the HAT mechanism. The most used peroxyl radical generators are the water-soluble 2,2-azobis(2-amidinopropane) dihydrochloride (AAPH) and the lipid-soluble 2,2-azobis(2,4-dimethylvaleronitrile) (AMVN). In the emulsion-based AAPH assay, the presence of antioxidant inhibits or slows down the oxidation of linoleic acid induced by peroxyl radicals [22]. The observed data on the antioxidant activity of all compounds are shown in Table 2.

**Table 2:** Antioxidant activity of test compounds.

Compound	IC_50_ (μM)	Compound	IC_50_ (μM)
1	2.01	10	3.29
5	1.09	14	3.00
6	3.94	15	3.80
9	2.10	16	9.10
7	0.70	20	1.20
8	2.19	21	1.83

As previous results have shown [28], there is no direct correlation between the results from the DPPH assay and those from the AAPH assay. Compound **7** is the most potent of the tested compounds at inhibiting lipid peroxidation with an IC_50_ of 0.70 µM. In comparison, its radical scavenging potential is one of the weakest with an IC_50_ of 24.6 µM (Table 1). As for the structural patterns dictating the protective effect against lipid peroxidation, one feature is apparent. The simultaneous absence of the ethylene moiety and the presence of a direct link of the carboxyl on the aromatic ring generates compounds with low antioxidant activity such as illustrated by DHB (**16**, IC_50_ = 9.10 µM). A consistent decrease of antioxidant activity is reported with derivatives of benzoic acid where the carbonyl is directly linked to the aromatic ring compared to derivatives of cinnamic acid [15]. The importance of the phenethyl ring has been conclusively demonstrated in the case of NF-κB inhibition by Lee and coworkers [29]. The most active compound among the CAPE analogues used in that study was the one bearing the phenethyl group [29]. 

Spacing the carboxyl with one C atom induced a 3-fold increase in potency as shown with DOPAC (compound **10**, IC_50_ = 3.29 µM). Also the presence of an ester group (phenethyl/phenpropionyl) has similar effects even when the carbonyl is directly linked to the aromatic ring. Compounds **20** and **21** with respective IC_50_ values of 1.2 and 1.83 µM show a 4-fold increase in potency compared to DHB (compound **16**), even if their carbonyl group is linked directly to the catechol ring.

The presence of the ethylene moiety is not a mandatory requisite for antioxidant activity. Caffeic acid (**1**) and its dehydrogenated counterpart dihydrocaffeic acid (**9**) have similar IC_50_ values: 2.01 and 2.10, respectively. This feature is also illustrated by the pairs CAPE (**5**) and **7** (IC_50_ = 1.09 and 0.70, respectively) and compounds **6** and **7** (IC_50_ = 3.94 and 2.19, respectively). Increasing the length of the linker by 1 C atom between the non-catechol aromatic ring and the carbonyl group consistently decreases the antioxidant activity. Compounds with an ethyl linker such as CAPE (**5**), **7**, **14** and **20** (IC_50_ = 1.09, 0.70, 3, 1.2 respectively) have better activities than their parent compounds with a propyl linker, namely **6**, **8**, **15** and **21** (IC_50_ = 3.94, 2.19, 3.80, 1.83 respectively).

## 3. Experimental

### 3.1. General

All chemicals used were purchased from Aldrich. Purification of compounds was carried out by silica gel circular chromatography (Chromatotron^®^, model 7924, Harrison Research, Palo Alto, CA, USA). Thin layer chromatography (TLC) was run on silica gel coated aluminum sheets (SiliaPlate TLC, Silicycle^®^) with detection by UV light (254 nm, UVS-11, Mineralight^®^ shortwave UV lamp). Melting points were obtained using a MEL-TEMP^®^ (model 1001D) melting point apparatus. FTIR spectra were recorded on a Nicolet^®^ Impact 400 spectrometer. NMR spectra were recorded on a Bruker^®^ Avance III 400 MHz spectrometer. High resolution mass measurements were performed on a Bruker^®^ Doltonics’ micrOTOF instrument in positive or negative electrospray. 

### 3.2. Synthesis

*(E)-3-Phenylpropyl-3-(3,4-Diacetoxyphenyl)acrylate* (**4**). A mixture of diacetylcaffeic acid (**2**, 300 mg, 1.13 mmol), thionyl chloride (5 mL) and two drops of DMF was heated at reflux for 4 h. The excess thionyl chloride (SOCl_2_) was removed on a rotovap, and the residue was dissolved in dry CH_2_Cl_2_ (4 mL). To this solution was slowly added pyridine (1 mL) and 3-phenylpropan-1-ol (232.0 μL, 1.71 mmol, 1.3 eq.). The resulting mixture was stirred overnight at room temperature. After removal of solvents, the residue was dissolved in dichloromethane (25 mL), the organic extract was washed with water (2 × 20 mL), brine (2 × 20 mL), and then dried over MgSO_4_. The resulting residue was purified by silica gel circular chromatography using 5–15% EtOAc-hex to afford 314 mg (yield = 73%) of the desired derivative **4** as a white solid. M.p. = 66–67 °C; Rf = 0.60 (30% EtOAc–hex); ^1^H-NMR (CDCl_3_, 25 °C); δ (ppm): 7.63 (d, *J* = 16.01 Hz, 1H, =CHC_ar_), 7.45–7.21 (m, 8H, H_ar_), 6.41 (d, 15.97 Hz, 1H, =CHCO), 4.25 (t, *J* = 6.52 Hz, 2H, CH_2_(CH_2_)_2_Ph), 2.77 (t, *J* = 7.44 Hz, 2H, (CH_2_)_2_CH_2_Ph), 2.33 (s, 6H, 2 × OAc), 2.06 (quint., *J* = 6.52 Hz, 2H, CH_2_CH_2_CH_2_Ph); ^13^C-NMR (CDCl_3_, 25 °C); δ (ppm): 168.08, 168.00, 166.60, 143.48, 142.76, 142.45, 141.20, 133.33, 128.47, 128.44, 126.41, 126.03, 123.93, 122.73, 119.34, 64.06, 32.26, 30.27, 20.66, 20.63; HRMS *m/z* calc. for C_22_H_22_O_6_ + (Na^+^) : 405.1309; found: 405.1311.

*(E)-3-Phenylpropyl-3-(3,4-dihydroxyphenyl)acrylate* (**6**). To a solution of **4** (165.81 mg, 0.43 mmol) in a 1:1 mixture of MeOH and CH_2_Cl_2_ (5 mL) was added guanidine·HCl (123 mg, 1.29 mmol, 1.5 eq. per OAc). The reaction mixture was stirred for 2 h. After removal of solvents, the residue was dissolved in EtOAc (25 mL), washed with saturated NH_4_Cl solution, with brine and dried over MgSO_4_. After filtration and evaporation of the solvent, pure deprotected **6** (123 mg, 93%) was obtained as a yellow solid. M.p. = 116–118 °C; Rf = 0.46 (5% MeOH–CH_2_Cl_2_); ^1^H-NMR (DMSO-*d_6_*, 25 °C): δ (ppm): 7.47 (d, *J* = 15.89 Hz, 1H, =CHC_ar_), 7.31–7.18 (m, 5H, H_ar_), 7.06–7.00 (m, 2H, H_ar_), 6.77 (d, *J* = 8.08 Hz, 1H, H_ar_), 6.28 (d, 15.89 Hz, 1H, =CHCO), 4.11 (t, *J* = 6.28 Hz, 2H, CH_2_(CH_2_)_2_Ph), 2.68 (t, *J* = 7.36 Hz, 2H, (CH_2_)_2_CH_2_Ph), 1.96–1.91 (m, 2H, CH_2_CH_2_CH_2_Ph); ^13^C-NMR (CDCl_3_, 25 °C) δ (ppm): 168.11, 168.02, 166.61, 143.46, 142.77, 142.43, 141.21, 133.33, 128.47, 128.45, 126.43, 126.04, 123.94, 122.73, 119.33, 64.06, 32.25, 30.27, 20.68, 20.65; HRMS *m/z* calc. for C_18_H_18_O_4_ + (Na^+^): 321.1097; found: 321.1083.

*Phenethyl-3-(3,4-dihydroxyphenyl)propanoate* (**7**). Compound **5** (152 mg, 0.53 mmol) was dissolved in MeOH (10 mL) and 10% Pd/C (16 mg) was added. The flask was sealed with a rubber septum and positive pressure of H_2_ was maintained by a balloon attached via a syringe needle. The progress of the reaction was monitored by TLC until the complete conversion after 16 h. The reaction mixture was filtered through celite and the solvent was removed under reduced pressure to give pure **7** (150 mg, quantitative yield) of a colorless oil. Rf: 0.23 (30% EtOAc–hex); ^1^H-NMR (CDCl_3_, 25 °C); δ (ppm): 7.34–7.21 (m, 5H, H_ar_), 6.77 (d, *J* = 8.00 Hz, 1H, H_ar_), 6.66 (s, 1H, H_ar_), 6.59 (d, *J* = 8.00 Hz, 1H, H_ar_), 4.31 (t, *J* = 7.00 Hz, 2H, CH_2_CH_2_Ph), 2.94 (t, *J* = 6.96 Hz, 2H, CH_2_CH_2_Ph)), 2.82 (t, *J* = 7.52 Hz, 2H, C_ar_CH_2_CH_2_C(=O)), 2.59 (t, *J* = 7.68 Hz, 2H, C_ar_CH_2_CH_2_C(=O)); ^13^C-NMR (CDCl_3_, 25 °C); δ (ppm): 173.49, 143.59, 142.08, 137.78, 133.25, 128.93, 128.52, 126.60, 120.57, 115.37, 65.16, 36.15, 35.04, 30.24; HRMS *m/z* calc. for C_17_H_18_O_4_ + (Na^+^): 309.1097; found: 309.1083.

*3-Phenylpropyl-3-(3,4-dihydroxyphenyl)propanoate* (**8**). Compound **8** was prepared from **6** (112 mg, 0.37 mmol) and 10% Pd/C (12 mg) following the same protocol as for **7** which gave **8** (110 mg, quantitative yield) as a yellow oil; Rf: 0.76 (30% EtOAc–hexanes); ^1^H-NMR (CDCl_3_, 25 °C); δ (ppm): 7.31–7.15 (m, 5H, H_ar_), 6.79–6.76 (m, 2H, H_ar_), 6.58 (d, *J* = 6.72 Hz, 1H, H_ar_), 4.08 (t, *J* = 6.09 Hz, 2H, CH_2_(CH_2_)_2_Ph), 2.78 (m, 2H, C_ar_CH_2_CH_2_C(=O)), 2.66–2.54 (m, 4H, C_ar_CH_2_CH_2_C(=O), (CH_2_)_2_CH_2_Ph)), 1.96–1.91 (m, 2H, CH_2_CH_2_CH_2_Ph); ^13^C-NMR (CDCl_3_, 25 °C); δ (ppm): 173.68, 143.26, 141.69, 141.12, 133.52, 128.45, 128.38, 126.02, 120.74, 115.44, 64.16, 36.03, 32.09, 30.22, 30.07; HRMS *m/z* calc. for C_18_H_20_O_4_ + (Na^+^): 323.1254; found: 323.1261.

*2-(3,4-Diacetoxyphenyl)acetic acid* (**11**). In a 10-mL round-bottom flask, 2-(3,4-dihydroxyphenyl) acetic acid (**10**, 496 mg, 2.95 mmol) was dissolved in acetic anhydride (3.5 mL) and sulfuric acid (0.1 mL) with stirring. The reaction mixture was stirred for 5 min and then ether (5 mL) was added. The solution was stirred for 24 h under argon. Subsequently, the mixture was poured into ice water (50 mL), extracted with EtOAc (50 mL), dried over MgSO_4_ and charcoal, filtered and concentrated. After recrystallization from EtOAc–hex (3:2), 2-(3,4-diacetyloxy)phenyl) acetic acid (**11**, 507 mg, 68%) was obtained as a white solid. M.p. = 104–106 °C; Rf = 0.37 (5% EtOAc-hex); ^1^H-NMR (CDCl_3_, 25 °C); δ (ppm): 7.22–7.16 (m, 3H, H_ar_), 3.66 (s, 2H, CH_2_), 2.31 (s, 6H, 2 × OAc); ^13^C-NMR (CDCl_3_, 25 °C); δ (ppm): 176.41, 168.25, 168.22, 142.00, 141.36, 131.88, 127.62, 124.47, 123.50, 40.18, 20.65, 20.63; HRMS *m/z* calc. for C_12_H_12_O_6_ (H^+^): 251.0561; found: 251.0565.

*Phenethyl-2-(3,4-diacetoxyphenyl)acetate* (**12**). Compound **12** was prepared from **11** (478 mg, 1.9 mmol), SOCl_2_ (12 mL), 2-phenylethan-1-ol (296 μL, 2.47 mmol), and pyridine (170 μL, 2.10 mmol) using the procedure for the synthesis of **4**. Purification by silica gel circular chromatography (5%–10% EtOAc–hex) gave **12** (467 mg, 69%) as a yellow oil. Rf = 0.36 (5% EtOAc-hex); ^1^H-NMR (400 MHz, CDCl_3_, 25 °C); δ (ppm): 7.33–7.25 (m, 4H, H_ar_), 7.19–7.18 (m, 2H, H_ar_), 7.14 (m, 2H, H_ar_), 4.34 (t, *J* = 6.96 Hz, 2H, CH_2_CH_2_Ph), 3.60 (s, 2H, C_ar_CH_2_CO), 2.95 (t, *J* = 6.96 Hz, 2H, CH_2_CH_2_Ph), 2.31 (s, 6H, 2 × OAc); ^13^C-NMR (101 MHz, CDCl_3_, 25 °C); δ (ppm): 170.68, 168.22, 168.14, 141.94, 141.17, 137.63, 132.62, 128.91, 128.50, 127.48, 126.56, 124.34, 123.38, 65.51, 40.65, 35.00, 20.66, 20.64; HRMS *m/z* calc. for C_20_H_20_O_6_ + (Na^+^): 379.1152; found: 379.1137.

*3-Phenylpropyl-2-(3,4-diacetoxyphenyl)acetate* (**13**). Compound **13** was prepared from **11** (500 mg, 1.9 mmol), SOCl_2_ (12 mL), 3-phenylpropan-1-ol (360 μL, 2.65 mmol), and pyridine (170 μL, 2.10 mmol) using the procedure for the synthesis of **4**. Purification by silica gel circular chromatography (5%–12% EtOAc–hex) gave **13** (491 mg, 67%) as a yellow oil. Rf = 0.64 (30% EtOAc-hex); ^1^H-NMR (CDCl_3_, 25 °C); δ (ppm): 7.34–7.30 (m, 2H, H_ar_), 7.25–7.18 (m, 6H, H_ar_), 4.15 (t, *J* = 6.48 Hz, 2H, CH_2_(CH_2_)2Ph), 3.63 (s, 2H, C_ar_CH_2_CO), 2.69 (t, *J* = 7.52 Hz, 2H, (CH_2_)_2_CH_2_Ph), 2.31 (s, 6H, 2 × OAc), 1.99 (quint., *J* = 6.60 Hz, 2H, CH_2_CH_2_CH_2_Ph); ^13^C-NMR (CDCl_3_, 25 °C): δ (ppm): 170.83, 168.23, 168.16, 142.03, 141.24, 141.13, 132.81, 128.57, 128.48, 128.45, 128.41, 127.48, 126.05, 124.37, 123.43, 64.40, 40.66, 32.12, 30.12, 20.64, 20.63; HRMS *m/z* calc. for C_21_H_22_O_6_ + (Na^+^): 393.1309; found: 393.1321.

*Phenethyl-2-(3,4-dihydroxyphenyl)acetate* (**14**). Compound **14** was prepared from **12** (205 mg, 0.57 mmol) and guanidine·HCl (183 mg, 1.9 mmol) following the same protocol as for **6**. Purification by silica gel circular chromatography (3%–10% EtOAc–hex) gave **14** (98 mg, 63%) as a yellow oil. Rf = 0.52 (5% MeOH–CH_2_Cl_2_); ^1^H-NMR (MeOD, 25 °C); δ (ppm): 7.27–7.14 (m, 5H, H_ar_), 6.72–6.70 (m, 2H, H_ar_), 6.54 (dd, *J* = 8.04 Hz, 1.76 Hz, 1H, H_ar_), 4.27 (t, *J* = 6.80 Hz, CH_2_CH_2_Ph), 3.44 (s, 2H, C_ar_CH_2_CO), 2.89 (t, *J* = 6.76 Hz, 2H, CH_2_CH_2_Ph); ^13^C-NMR (MeOD, 25 °C); δ (ppm): 172.56, 144.91, 144.06, 137.91, 128.59, 128.04, 126.05, 125.51, 120.28, 116.04, 114.92, 65.13, 40.14, 34.60; HRMS *m/z* calc. for C_16_H_16_O_4_ + (Na^+^): 295.0941; found: 295.0932.

*3-Phenylpropyl-2-(3,4-dihydroxyphenyl)acetate* (**15**). Compound **15** was prepared from **13** (295 mg, 0.79 mmol), guanidine·HCl (254 mg, 2.66 mmol), and Et_3_N (1.1 mL) following the same protocol as for **6**. Purification by silica gel circular chromatography (5%–8% EtOAc–hex) gave **15** (136 mg, 60%) as a yellow oil. Rf = 0.47 (5% MeOH–CH_2_Cl_2_); ^1^H-NMR (MeOD, 25 °C); δ (ppm): 7.27–7.23 (m, 2H, H_ar_), 7.18–7.11 (m, 3H, H_ar_), 6.76–6.72 (m, 2H, H_ar_), 6.62 (dd, *J* = 8.08 Hz, 1.96 Hz, 1H, H_ar_), 4.06 (t, *J* = 6.32 Hz, 2H, CH_2_(CH_2_)_3_Ph), 3.47 (s, 2H, C_ar_CH_2_CO), 2.63 (t, *J* = 7.44 Hz, 2H, (CH_2_)_3_CH_2_Ph), 1.92 (quint., *J* = 6.44 Hz, 4H, CH_2_(CH_2_)_2_CH_2_Ph); ^13^C-NMR (MeOD, 25 °C); δ (ppm): 172.70, 145.00, 144.30, 141.14, 128.08, 127.99, 125.68, 125.52, 120.23, 115.95, 114.92, 53.56, 40.23, 31.54, 30.05; HRMS *m/z* calc. for C_17_H_18_O_4_ + (Na^+^): 309.1097; found: 309.1085.

*3,4-Diacetoxybenzoic acid* (**17**). Compound **17** was prepared from 3,4-dihydroxybenzoic acid (DHB, **16**, 1 g, 6.52 mmol), acetic anhydride (6.5 mL), sulfuric acid (0.1 mL), and ether (20 mL) following the same protocol as for **11**. Recrystallization from EtOAc–hex (3:2), acid 3,4-bis(acetyloxy)benzoic acid (**17**, 1.3 g, 83%) was obtained as a white solid. M.p. = 158–160 °C; Rf = 0.32 (30% EtOAc–hex); ^1^H-NMR (CDCl_3_, 25 °C): δ (ppm): 11.42 (s large, 1H, OH), 8.05 (dd, *J* = 8.48 Hz, 1.88 Hz, 1H, H_ar_), 7.97 (d, *J* = 1.84 Hz, 1H, H_ar_), 7.35 (d, *J* = 8.48 Hz, 1H, H_ar_), 2.35 (s, 6H, 2 × OAc); ^13^C-NMR (CDCl_3_, 25 °C); δ (ppm): 170.32, 167.97, 167.63, 146.73, 142.10, 128.78, 127.77, 125.71, 123.68, 20.69, 20.56; HRMS *m/z* calc. for C_11_H_10_O_6_ (H^+^): 237.0405; found: 237.0404.

*Phenethyl-3,4-diacetoxybenzoate* (**18**). Compound **18** was prepared from **17** (505 mg, 2.1 mmol), SOCl_2_ (12 mL), 2-phenylethan-1-ol (340 μL, 2.84 mmol), and pyridine (200 μL, 2.47 mmol) using the procedure for the synthesis of **4**. Purification by silica gel circular chromatography (5%–10% EtOAc–hex) gave **18** (486 mg, 67%) as a yellow oil. Rf = 0.52 (30% EtOAc–hex); ^1^H-NMR (CDCl_3_, 25 °C): δ (ppm): 7.92–7.88 (m, 2H, H_ar_), 7.38–7.31 (m, 3H, H_ar_), 7.28–7.25 (m, 3H, H_ar_) 4.32 (t, *J* = 6.5 Hz, CH_2_CH_2_Ph), 2.78 (t, *J* = 7.7 Hz, 2H, CH_2_CH_2_Ph), 2.32 (s, 6H, 2 × OAc); ^13^C-NMR (CDCl_3_, 25 °C); δ (ppm): 168.01, 167.83, 146.82, 143.01, 140.12, 128.84, 128.71, 128.36, 128.06, 126.12, 125.02, 123.65, 64.78, 34.41, 20.72, 20.43; HRMS *m/z* calc. for C_19_H_18_O_6_ + (Na^+^): 365.0996; found: 365.0996.

*3-Phenylpropyl-3,4-diacetoxybenzoate* (**19**). Compound **19** was prepared from **17** (500 mg, 2.1 mmol), SOCl_2_ (15 mL), 3-phenylpropan-1-ol (380 μL, 2.79 mmol), and pyridine (180 μL, 2.23 mmol) using the procedure for the synthesis of **4**. Purification by silica gel circular chromatography (1%–18% EtOAc–hex) gave **19** (493 mg, 66%) as a yellow oil. Rf = 0.48 (30% EtOAc–hex); ^1^H-NMR (CDCl_3_, 25 °C); δ (ppm): 7.95 (dd, *J* = 8.44, 2 Hz, 1H, H_ar_), 7.86 (d, *J* = 2 Hz, 1H, H_ar_), 7.31–7.28 (m, 3H, H_ar_), 7.24–7.22 (m, 3H, H_ar_) 4.36 (t, *J* = 6.5 Hz, CH_2_CH_2_CH_2_Ph), 2.80 (t, *J* = 7.8 Hz, 2H, CH_2_CH_2_CH_2_Ph), 2.34 (s, 6H, 2 × OAc), 2.12 (quint., *J* = 6.5 Hz, 2H, CH_2_CH_2_CH_2_Ph); ^13^C-NMR (CDCl_3_, 25 °C); δ (ppm): 168.03, 167.72, 145.94, 141.98, 141.07, 128.97, 128.50, 128.44, 128.11, 126.07, 124.99, 123.49, 64.71, 32.29, 30.19, 20.69, 20.59; HRMS *m/z* calc. for C_20_H_20_O_6_ + (Na^+^): 379.1152; found: 379.1134.

*Phenethyl-3,4-dihydroxybenzoate* (**20**). Compound **20** was prepared from **18** (250 mg, 0.73 mmol), guanidine·HCl (246 mg, 2.57 mmol), and Et_3_N (1.1 mL) following the same protocol as for **6**. Purification by silica gel circular chromatography (5%–10% EtOAc–hex) gave **20** (170 mg, 90%) as a yellow solid. M.p. = 128–130 °C; Rf = 0.82 (5% MeOH–CH_2_Cl_2_); ^1^H-NMR (MeOD, 25 °C); δ (ppm): 7.42–7.39 (m, 2H, H_ar_), 7.31–7.30 (m, 4H, H_ar_), 7.25–7.19 (m, 1H, H_ar_), 6.80 (d, *J* = 8.12 Hz, 1H, H_ar_), 4.47–4.43 (m, 2H, CH_2_CH_2_Ph), 3.07–3.02 (m, 2H, CH_2_CH_2_Ph); ^13^C-NMR (MeOD, 25 °C); δ (ppm): 166.85, 150.32, 144.76, 138.14, 128.60, 128.11, 126.12, 122.22, 121.30, 116.00, 114.42, 65.01, 34.83; HRMS *m/z* calc. for C_15_H_14_O_4_ + (Na^+^): 281.0784; found: 281.0774.

*3-Phenylpropyl-3,4-dihydroxybenzoate* (**21**). Compound **21** was prepared from **19** (150 mg, 0.42 mmol), guanidine·HCl (133 mg, 1.4 mmol), and Et_3_N (578 μL) following the same protocol as for **6**. Purification by silica gel circular chromatography (5%–10% EtOAc–hex) gave **21** (103 mg, 90%) as a yellow solid. M.p. = 125–126 °C; Rf = 0.7 (5% MeOH–CH_2_Cl_2_); ^1^H-NMR (DMSO-*d_6_*, 25 °C); δ (ppm): 7.41–7.39 (m, 1H, H_ar_), 7.35–7.27 (m, 3H, H_ar_), 7.24–7.17 (m, 3H, H_ar_), 6.83 (d, *J* = 8.2 Hz, 1H, H_ar_), 4.17 (t, *J* = 6.4 Hz, 2H, CH_2_CH_2_CH_2_Ph), 2.71 (t, *J* = 7.5 Hz, 2H, CH_2_CH_2_CH_2_Ph), 1.98 (quint., *J* = 6.4 Hz, 2H, CH_2_CH_2_CH_2_Ph); ^13^C-NMR (DMSO-d6, 25 °C); δ (ppm): 166.15, 150.88, 145.53, 141.63, 128.82, 128.78, 126.34, 122.27, 121.16, 116.74, 115.78, 63.75, 32.0, 30.36; HRMS *m/z* calc. for C_16_H_16_O_4_ + (Na^+^): 295.0941; found : 295.0935.

### 3.3. *In Vitro* Antioxidant Activity

#### 3.3.1. AAPH Assay

The antioxidant assay was performed as previously described by Liégeois and *al.* [30]. Briefly, a 5 mM phosphate-buffered solution (pH 7.4) containing 0.05% Tween 20 (Sigma-Aldrich) and 0.16 mM linoleic acid (Cayman Chemical, Ann Arbor, MI, USA) was preheated at 40 °C. Test compounds or their diluent (DMSO) were added to the mix at the indicated concentrations. The oxidation reaction, performed under a constant temperature of 37 °C, was initiated with the addition of 50 µL of 2,2'-azobis(2-amidinopropane) dihydrochloride (AAPH) solution (10 mg·mL^−1^) (Cayman Chemical) to 1 mL of the above solution. The rate of lipid oxidation was determined by measuring the absorbance at 234 nm with a Thermo Varioskan UV visible spectrophotometer at every 5 min for 3 h. Inhibition of linoleic acid oxidation was calculated as follows: (%) = (1 − rate absorbance change with test compound/rate of absorbance change with solvent control) × 100.

#### 3.3.2. DPPH Assay

The radical scavenging activity of test compounds was measured as previously described using 2,2-diphenyl-1-picrylhydrazyl (DPPH) as a stable radical [31] with slight modifications. Particular care was taken in the preparation of the control (DPPH reagent + ethanol as a diluent without test compounds). Controls with O.D. of 0.350–0.360 at 520 nm were deemed as acceptable to avoid variations in IC_50_ calculations. DPPH in ethanol (1 mL, 60 mM) was mixed with the test compounds (1 mL) at the indicated concentrations or their diluent (ethanol). Each mixture was then shaken vigorously and held in the dark for 30 min at room temperature. The absorbance of DPPH at 520 nm was then measured. The radical scavenging activity was expressed in terms of % inhibition of DPPH absorbance:

% Inhibition = [(Acontrol − Atest)/Acontrol)] × 100

where Acontrol is the absorbance of the control (DPPH solution without test compound) and Atest is the absorbance of the test sample (DPPH solution plus compound). 

#### 3.3.3. Data Analysis

All data are expressed as means of two experiments; each experiment being performed in triplicate. IC_50_ values were calculated from a sigmoidal concentration-response curve-fitting model with a variable slope on GraphPad Prism 5 software (GraphPad Software, San Diego, CA, USA).

## 4. Conclusions

Structural modifications of the CAPE (**5**) core have shown the possibility to synthesize molecules or analogues with improved antioxidant/radical scavenging properties when compared to CAPE (**5**). Well-known structural prerequisites include the presence of a catechol ring and a short spacer between the catechol and the carbonyl moiety. In our series of CAPE (**5**) analogues, the presence of the ethylene moiety was important, but not a determining factor for radical scavenging using an ET mechanism while in a HAT type mechanism (AAPH assay), its presence mattered less. As to the importance of the linker between the phenyl and the carbonyl, it appears that the ethyl promoted better antioxidant activity compared to the propionyl group.
